# Metastases and Primary Brain Tumors Affecting the Fornix of the Brain

**DOI:** 10.7759/cureus.57612

**Published:** 2024-04-04

**Authors:** Abidin Emre Kılıç, Ezel Yaltırık Bilgin, Özkan Ünal

**Affiliations:** 1 Radiology, Dr. Abdurrahman Yurtaslan Ankara Oncology Training and Research Hospital, Ankara, TUR

**Keywords:** metastasis, limbic lobe, neoplasms, magnetic resonance imaging, fornix brain

## Abstract

Background

The aim of this study is to evaluate the clinical and radiological findings of metastatic tumors and primary brain tumors affecting the fornix.

Methods

Between January 2015 and March 2023, we retrospectively evaluated 1087 patients of both sexes who underwent cranial magnetic resonance imaging (MRI) for a preliminary diagnosis of intracranial malignancy in the radiology department of our hospital. Two radiologists with six and 10 years of experience in MRI examination assessed the relationship between primary and metastatic tumors and the fornix.

Results

Involvement of the fornix was diagnosed in 29 of the 1087 patients (2.66%), of which fornix was affected by metastatic lesions in 14 patients (48.2%) and primary tumors in 15 patients (51.7%). The majority of metastatic lesions were from lung and breast cancers, with other tumor types including osteosarcoma, renal cell carcinoma, pancreatic adenocarcinoma, pleomorphic sarcoma, and diffuse large B-cell lymphoma. Among all primary tumors, glioblastoma was the most common primary brain tumor invading the fornix, with other diagnoses including diffuse astrocytoma, medulloblastoma, and anaplastic oligodendroglioma. Metastatic and primary brain tumors affecting the fornix were detected over a broad timeline, from the time of diagnosis up to 120 months after diagnosis. A retrospective evaluation of medical records revealed memory deficits in four patients.

Conclusion

The fornix can be affected by both metastatic and primary brain tumors. It is crucial to understand the relevant neuroanatomical relationships when evaluating lesions that affect the fornix.

## Introduction

The limbic lobe is a complex system comprising cortical and subcortical structures. Key structures of the limbic system include the hippocampus, parahippocampus, cingulate gyrus, amygdala, and hypothalamus [1]. The fornix, a connection pathway within the limbic system, is one of the commissural pathways that ensure the relationship between the cerebral hemispheres. The fornix has a semicircular morphology extending from anterior to posterior; its structure comprises four anatomical components: the columns, body, crura, fimbriae, and alveus. The fimbriae and alveus are fused with the hippocampus and extend medially to the temporal horn of the lateral ventricle. During their course, the crura and body are situated in close proximity to the septum pellucidum and internal cerebral veins. The anteriorly located column of the fornix joins with the corpus mamillare [2,3].

Technical advances in neuroimaging have allowed detailed evaluation of the limbic system and fornix. The most important advances in the evaluation of the anatomy and morphology of the limbic system have been in magnetic resonance imaging (MRI). Along with the multi-plane capability and high resolution of MRI, diffusion-weighted and diffusion-tensor imaging techniques have led to a better understanding of the diseases affecting the limbic system [4,5].

The limbic system can be affected by a wide variety of malignant and non-malignant diseases. This critical review delves into the intricacies of fornix involvement in intracranial tumors, aiming to assess the strengths and limitations of existing research while identifying avenues for improvement. Understanding the role of the fornix in cognitive function and its implications for patient management is crucial for enhancing clinical care in neuro-oncology. This article was previously posted to the Research Square preprint server on September 8, 2023.

## Materials and methods

Ethical considerations

Approval for the study (2023-04/38) was obtained from the local ethics committee. Patients provided informed consent before receiving contrast-enhanced brain MRI scans. An informed consent form for this study was not requested from the patients due to the retrospective nature of the study.

Patient selection

Between January 2015 and March 2023, a retrospective evaluation was conducted among 1087 patients of both sexes, aged 18-99 years, who underwent cranial MRI at our hospital’s radiology department for preliminary diagnosis of intracranial malignancy. Two radiologists, with six and 10 years of experience in MRI examination, assessed the relationship between primary and metastatic tumors with the fornix. A neuroradiologist with 20 years of experience was consulted to decide inclusion/exclusion when there was uncertainty about the relationship of the tumors with the fornix.

Patients with primary and metastatic tumors involving the fornix were included in the study, and patients whose MRI scan was not of diagnostic quality and whose fornix was not involved were excluded.

MRI evaluations

MRI studies were conducted with a high-field system (1.5T, SIGNA Explorer GE Healthcare, USA) with a dedicated 16-channel head-neck coil. MRI data were acquired using the following sequences: (1) axial T1-weighted imaging sequence with TE/TR, 8/509 msec; slice thickness, 4 mm; matrix size, 224 × 224; FOV, 220 × 220 mm^2^; (2) axial T2-weighted imaging sequence with TE/TR, 127/4082 msec; slice thickness, 4 mm; matrix size, 416 × 416; FOV, 220 × 220 mm^2^; (3) sagittal T2-weighted imaging sequence with TE/TR, 114/5638 msec; slice thickness, 4 mm; matrix size, 352 × 352; FOV, 240 × 240 mm^2^; (4) coronal T2* GRE weighted imaging sequence with TE/TR, 18/660 msec; slice thickness, 5 mm; matrix size, 320 × 192; FOV, 230 × 230 mm^2^; (5) axial fluid-attenuated inversion recovery (FLAIR) sequence with TE/TR, 104/9000 msec; slice thickness, 4 mm; matrix size, 288 × 192; FOV, 220 × 220 mm^2^; (6) axial diffusion weighted images with TE/TR, 93/7702 msec; slice thickness, 4 mm; matrix size, 160 × 160; FOV, 220 × 220 mm^2^; (7) axial post-contrast T1-weighted imaging sequence with TE/TR, 8/509 msec; slice thickness, 4 mm; matrix size, 224 × 224; FOV, 220 × 220 mm^2^; (8) post-contrast 3D sagittal T1 cube sequence with TE/TR, 13/502 msec; slice thickness, 1.2 mm; matrix size, 320 × 192; FOV, 256 × 256 mm^2^; (9) coronal FLAIR sequence with TE/TR, 81/9000 msec; slice thickness, 3 mm; matrix size, 320 × 192; FOV, 240 × 240 mm^2^.

Post-contrast thin-sliced sagittal, axial, and coronal images obtained from 3D sequences were preferred for the evaluation of fornix lesions. The presence of enhancement on post-contrast T1-weighted images was determined as a criterion for fornix involvement. However, hyperintensity on FLAIR images in gliomas without contrast enhancement was considered as fornix involvement.

## Results

Fornix involvement was diagnosed in 29/1087 (2.66%) patients (18 males and 11 females; age range, 22-79 years) after retrospective evaluation of cranial MRI scans. The lesions were classified into two main groups: primary and metastatic lesions. 

Assessment of metastatic tumors

Of the 29 cases of fornix involvement, 14 (48.2%) were due to metastatic disease, with seven patients having metastatic lesions localized to the fornix and seven patients having metastatic lesions localized to the fornix with adjacent structures. The majority of primary tumors were lung and breast cancer, with other primary tumors including osteosarcoma, renal cell carcinoma, pancreatic adenocarcinoma, gluteal pleomorphic sarcoma, and diffuse large B-cell lymphoma. 

**Figure 1:**
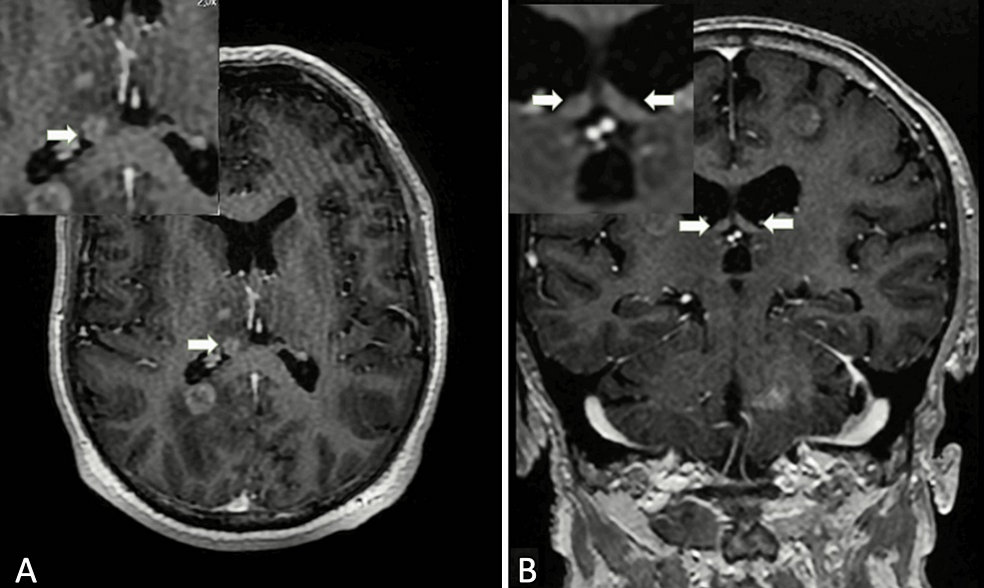
Metastatic lesions (A) Post-contrast axial T1-weighted image showing metastatic lesion localized in the right crus of the fornix (white arrows) in a 60-year-old male with a history of lung adenocarcinoma. (B) Post-contrast coronal T1-weighted image showing two metastatic lesions localized in the bilateral crus of the fornix in a 69-year-old male with a history of small-cell lung cancer (white arrows). 2.0× magnified images of the lesions are shown in the upper left corner.

Two patients had more than one fornix metastasis, with four metastases located on the crura (two on the right and two on the left) in the patient diagnosed with small-cell lung cancer and two metastases found on the left fornix in the patient with pancreatic adenocarcinoma. In a patient with lung adenocarcinoma, a single fornix lesion involved the bilateral body of the fornix, whereas other patients had a single fornix metastasis. Metastatic lesions were most commonly found in the posterior compartments of the fornix (Figure 1).

Metastatic lesions occurred over a wide time interval among the study sample, from the time of diagnosis to 120 months after diagnosis. All patients presented with other intracranial and distant metastases by the time fornix metastasis was identified. In the retrospective review of medical reports, one patient had a memory deficit; except for one patient, all patients had nonspecific clinical symptoms. Clinical and radiological features of the metastatic lesions are summarized in Table 1.

**Table 1:** Radiologic and clinical features of fornix metastases M, male; F, female; TOD, at the time of diagnosis; NSS, nonspecific symptoms; R, right; L, left; ER+, estrogen receptor-positive; PR+, progesterone receptor-positive; ALK, anaplastic lymphoma kinase mutation; EGFR, epidermal growth factor receptor mutation

Patient no.	Age(y); gender	Primary tumor; subtype	Lateralization; location	Identified time interval (month)	Distant metastasis	Symptom
1	60; M	Lung cancer; adenocarcinoma	R; crus	67	Contralateral lung, lymph node	NSS
2	61; F	Breast cancer; invasive ductal carcinoma ER+ PR+	R; body	6	Lung, liver, bone	Memory deficit
3	49; M	Lung cancer; adenocarcinoma ALK+	R, L; body	60	Lymph node	-
4	46; F	Breast cancer; invasive ductal carcinoma ER+ PR+	R; crus	13	Spinal leptomeningeal metastasis	NSS
5	69; M	Lung cancer; small-cell lung cancer	R, L; body, crus	TOD	Contralateral lung	-
6	64; M	Pancreas; adenocarcinoma	L; body, crus	10	Liver, bone, lymph node	-
7	51; M	Diffuse large B-cell lymphoma	L; crus	8	Lymph node involvement	-
8	54; F	Breast cancer; invasive ductal carcinoma ER+ PR+	L; crus-fimbria with hippocampus	23	Liver, lung, bone	-
9	56; F	Breast cancer; invasive ductal carcinoma ER+ PR+	L; crus-fimbria with parietooccipital lobe	48	Liver, lung, bone	-
10	57; M	Lung cancer; adenocarcinoma	L; crus with parietooccipital lobe	120	Bone	-
11	72; M	Lung cancer; adenocarcinoma ALK- EGFR-	L; crus-fimbria with thalamus	TOD	Adrenal, lymph node	NSS
12	79; M	Renal cell carcinoma; clear cell carcinoma	R; crus-fimbria with temporal lobe	115	Lung	-
13	22; M	Osteosarcoma	R; crus-fimbria with parietal lobe	19	Lung	-
14	49; F	Pleomorphic sarcoma	L; crus-fimbria with hypothalamus	67	Lung	NSS

However, all patients with fornix metastases also had supratentorial and infratentorial multiple metastatic lesions, and other organ and system metastases were detected. When metastatic lesions were evaluated completely, estrogen receptor positivity and progesterone receptor positivity were found with fornix metastasis in all patients with breast cancer, except for one medical record that could not be accessed.

Assessment of primary brain tumors

**Figure 2:**
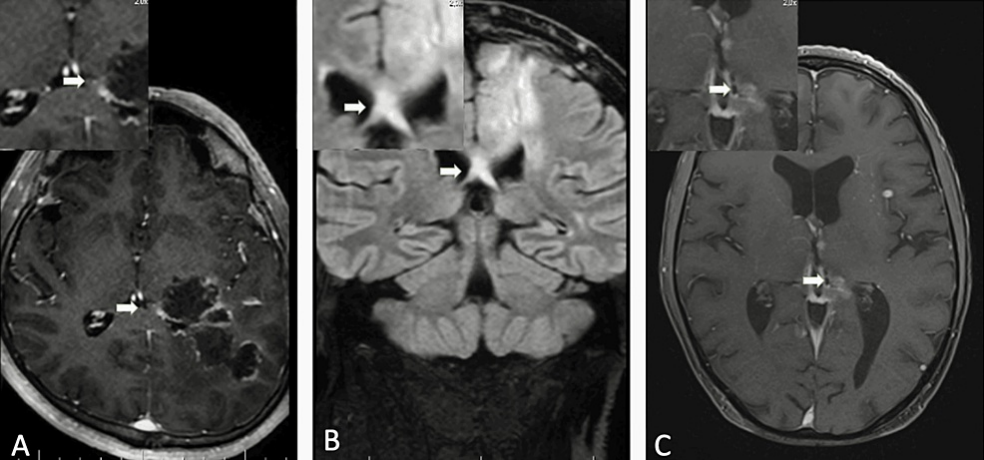
Primary tumors (A) Post-contrast T1-weighted image showing recurrent lesion invading the left crus of the fornix (arrow) in a 56-year-old male diagnosed with glioblastoma. (B) Post-contrast fluid-attenuated inversion recovery (FLAIR) image showing lesion crossing midline via the corpus callosum and invading the bilateral crus of the fornix (arrow) in a 45-year-old male diagnosed with diffuse astrocytoma. (C) Post-contrast T1-weighted image showing recurrent lesion invading both the crus and the body of the left fornix (arrow) in a 25-year-old male diagnosed with medulloblastoma. 2.0× magnified images of the lesions are shown in the upper left corner.

Fornix invasion was present in 15 patients (51.7%; age range, 25-78 years) diagnosed with a primary brain tumor. Glioblastoma was the most common primary brain tumor invading the fornix, with other diagnoses including diffuse astrocytoma, medulloblastoma, and anaplastic oligodendroglioma (Figure 2).

Fornix invasion was present in the MRI studies of eight patients at the time of diagnosis, whereas fornix invasion was detected in recurrent lesions in seven patients five to 91 months after diagnosis. Eight of the fornix-invading primary brain tumors crossed the midline. 

Memory deficit was found in three patients, and others had nonspecific symptoms. Clinical and radiological features of primary brain tumors that invaded the fornix are summarized in Table 2.

**Table 2:** Radiologic and clinical features of primary brain tumors that invade the fornix M, male; F, female; R, right; L, left; TOD, at the time of diagnosis; NSS, nonspecific symptoms

Patient no.	Age(y); gender	Primary tumor	Lateralization; location	Identified time interval (month)	Symptom	Crossing midline
1	52; M	Glioblastoma	R; crus-fimbria	TOD	NSS	+
2	52; M	Glioblastoma	L; crus-fimbria	Recurrent-10	Memory deficit	+
3	56; M	Glioblastoma	L; crus-fimbria	TOD	NSS	-
4	38; M	Glioblastoma	L; crus-fimbria	TOD	NSS	+
5	78; M	Glioblastoma	R, L; column and body	TOD	Memory deficit	+
6	56; M	Glioblastoma	L; crus-fimbria	TOD	NSS	-
7	57; F	Glioblastoma	L; column	Recurrent-26	NSS	+
8	64; M	Glioblastoma	R, L; column	Recurrent-24	NSS	+
9	55; F	Glioblastoma	L; crus-fimbria, body	Recurrent-22	NSS	-
10	50; F	Glioblastoma	R, L; column	Recurrent-5	NSS	+
11	53; F	Glioblastoma	L; crus-fimbria, body	Recurrent-13	Memory deficit	-
12	25; M	Medulloblastoma	L; crus-body	Recurrent-91	NSS	+
13	63; M	Anaplastic oligodendroglioma	R; crus-fimbria	Recurrent-14	-	+
14	45; M	Diffuse astrocytoma	R, L; crus-fimbria	TOD	-	+
15	69; F	Diffuse astrocytoma	L; crus-fimbria	TOD	NSS	-

## Discussion

The fornix may be affected in non-tumoral diseases (e.g., infection, multiple sclerosis, Wernicke’s encephalopathy, mesial temporal sclerosis, trauma-surgery, and infarction), with memory deficit as one possible clinical manifestation [6-11]. Lymphoma and glioblastoma, which are defined as midline tumors, are the most well-known tumors affecting the fornix. It has been argued that these lesions mainly involve corpus callosum involvement in the midline and subsequently spread to the fornix [12,13]. Diffuse astrocytoma is the other reported primary brain tumor that invades the fornix [14]. In this study, we included tumoral lesions affecting the fornix. Our results showed that 14 of 1087 (1.28%) patients had fornix metastasis, and 15 of 1087 (1.37%) patients were diagnosed with primary brain tumors that invade fornix.

In the current study, we showed that in addition to glioblastoma and lymphoma, diffuse astrocytoma, medulloblastoma, and oligodendroglioma can invade the fornix. Although it has been reported that the fornix can be involved in the spread of tumors that cross the midline [12], in the current study, the fornix was invaded by tumors located unilaterally in almost half of the patients diagnosed with primary brain tumors.

We found lesions that metastasized to the fornix and metastatic lesions that invaded the fornix with adjacent structures in 15 patients. To the best of our knowledge, this was the first study evaluating fornix metastasis in the English literature. The primary tumors metastasizing to the fornix originated from the lung, breast, pancreas, and kidney. Primary tumors constituted a more heterogeneous group in metastatic lesions invading the fornix. Metastatic lesions affecting the fornix were detected over a broad timeline, spanning at the time of diagnosis up to 120 months after diagnosis. 

Due to the thin formation and close association of the fornix with neighboring structures, it can sometimes be difficult to evaluate with imaging. However, neuroimaging findings and clinical symptom association provide important information about the function of the fornix. MRI examinations of the fornix and other components of the limbic system are frequently conducted in Alzheimer’s disease. [15]. In addition, volumetric examinations of the fimbria fornix have revealed the effect of the right fimbria on spatial memory [16]. When non-metastatic tumoral lesions were evaluated in the current study, one patient with a diagnosis of astrocytoma and one with glioblastoma had memory deficits at the time of diagnosis. In these patients, there was also the involvement of the fornix and other components of the limbic lobe. In one patient diagnosed with glioblastoma, at the time of diagnosis, another limbic-lobe-component invasion was present apart from the fornix, with memory deficit developing after left crus-fimbria invasion.

In addition to the importance of the fornix in cognitive functions and emotional effectiveness, it also has a critical role in memory. The function of the fornix has also become clearer as a result of neuroimaging findings with clinical correlation [17-19]. In our retrospective evaluation made from patient medical records, memory deficit was detected in one patient diagnosed with breast cancer metastasizing to the right fornix body, whereas nausea, vomiting, and headache were found in four patients, with these symptoms nonspecific to the fornix. However, although information covering disease progression and regression was included in the medical records, symptoms were not included except for four patients with fornix involvement. This situation constitutes the most important limitation of our study. Specifically, lesions that metastasize to the fornix were found with multiple brain metastases; the clinical findings of these metastatic lesions may not have been recorded, as they may have overshadowed the memory-related findings.

In our study, we found hormone receptor positivity in patients with breast cancer metastasizing to the fornix, with the exception of one patient whose medical record was inaccessible. Apart from this finding, no common histopathological finding was found in other patient groups.

It should be noted that MRI evaluation of the fornix isn’t easy due to its thin structure and its close proximity to adjacent structures; post-contrast thin-slice images, preferably obtained from 3D sequences, provided great convenience for the anatomical identification of the fornix [20]. The continuity of the fornix and its relationship with neighboring structures (e.g., internal cerebral veins) play an important role in anatomical localization. However, focal enlargement of the internal cerebral vein mimicked fornix metastasis in one patient, who was subsequently excluded from the study. Thus, vascular enlargement may be a potential pitfall in MRI evaluation of the fornix. Collaborative efforts between neuroimaging specialists, neurosurgeons, oncologists, and neuropsychologists could facilitate a holistic understanding of fornix involvement in intracranial tumors, leading to improved patient care and outcomes.

Our study has potential limitations. The study’s retrospective design and relatively small sample size may limit the generalizability of its findings. Future research with larger, more diverse cohorts could provide a more comprehensive understanding of fornix involvement in intracranial tumors. In addition, this study acknowledges the importance of follow-up imaging in assessing fornix involvement over time but does not provide longitudinal data on disease progression or treatment outcomes. Incorporating longitudinal studies could elucidate the natural history of fornix lesions and inform treatment strategies.

## Conclusions

In this study, we reported eight years of experience with the fornix, aiming to contribute to the literature on the fornix and limbic systems. The fornix can be affected by both metastatic and non-metastatic intracranial tumors. Although breast and lung cancers were the most common metastatic lesions, glioblastoma was the most common primary brain tumor invading the fornix. Invasion of the fornix was demonstrated in follow-up imaging and imaging at the time of diagnosis.

Knowing the neuroanatomical relationships facilitates the evaluation of lesions affecting the fornix. However, in the presence of a lesion affecting the fornix, accurate reporting of the lesion is important in the clinical correlation of cognitive function and memory deficit. Assessment of symptoms and imaging findings can both help understand the impact of the fornix on cognitive function.

There are opportunities for improvement, particularly in terms of methodological rigor and longitudinal data collection. By addressing these limitations and fostering multidisciplinary collaboration, future research can advance our understanding of fornix pathology and inform evidence-based approaches to patient management in neuro-oncology.
